# Rose-Coloured Ringworm Cultures

**Published:** 1909-02-06

**Authors:** 


### DERMATOLOGY.

### ROSE-COLOURED RINGWORM CULTURES.

The more fully cases of ringworm are investigated, the more clear does it become that the pathology of the disease is less simple than was at one time supposed, and that the number of different vegetable parasites that may produce "ringworm" is considerable. The chief importance of identifying the parasite as exactly as possible is in connection with tracing the source from which the patient caught it. This is impossible in the majority of cases on account of the expense, ringworm being a much commoner complaint amongst the poor than it is amongst the rich. In a well-to-do case, however, it is well worth while taking every step that may assist in determining whether the patient acquired the infection from another human being, or from a dog, cat, horse, donkey, calf, or bird. The great majority of ordinary cases of ringworm are due to transmission from one human being to another; but it would be wrong to assume that every case had been transmitted thus, particularly when there are any points about it that are in the least degree unusual. The variety of parasite can be detected with fair certainty if, in addition to staining and examining the infected hairs, cultivations are made upon suitable media. If it is found that the culture-type indicates infection from some animal, a dog for example, it may clearly be of the greatest importance to the household; the disease in the domestic animal may not have been suspected, and unless detected in this way it may continue to infect children and adults in an apparently mysterious way for a long time.

The most typical of all the ringworm parasites, from a cultural point of view, is that which Sabouraud described as the *Trichophyton ectothrix à cultures roses*. It is also referred to by French observers as the *Trichophyton rosaceum d'origine aviaire*. These two titles summarise the main points about it. Whereas almost all other ringworm parasites give cultures that are white, the variety which occurs in birds—poultry, canaries, pigeons—and thence infects man from time to time, is at once distinguished by the rose colour of its cultures.

